# Lidocaine for pain and discomfort relief during nasogastric tube insertion: A systematic review

**DOI:** 10.1016/j.ijnsa.2026.100710

**Published:** 2026-10-14

**Authors:** Femke van Kessel, Harm H.J. van Noort, Minke Reimink, Kim Kamphorst

**Affiliations:** a Scientific Office, Deventer Hospital, Nico Bolkesteinlaan 75, Deventer, 7416 SE, The Netherlands; b Centre for Abdominal and Pelvine Care, Radboud University Medical Centre, Geert Grooteplein Zuid 10, Nijmegen, 6525 GA, The Netherlands; c Department of Surgery, Radboud University Medical Centre, Geert Grooteplein Zuid 10, Nijmegen, 6525 GA, The Netherlands; d Oncologic Centre, Deventer Hospital, Nico Bolkesteinlaan 75, Deventer, 7416 SE, The Netherlands

**Keywords:** Nasogastric tube, Lidocaine, Procedural pain, Topical analgesics, Adults, Clinical nursing

## Abstract

**Background:**

Nasogastric tube (NGT) insertion is a common procedure in hospitalized adults, associated with pain and discomfort. Lidocaine has been studied to reduce procedural pain, but evidence on the most effective, clinically feasible approach remains limited.

**Objective:**

This review aimed to systematically review and synthesise evidence on the effectiveness and safety of topical anaesthetics or lubricants for reducing pain and discomfort during NGT insertion in adults.

**Method:**

A systematic search was conducted in MEDLINE, Cochrane Database of Systematic Reviews, CENTRAL, and CINAHL from inception to October 28, 2025. Randomized controlled trials (RCTs) in adults were eligible if they assessed non-nebulised topical anaesthetics or lubricants and reported pain, discomfort, procedure-related outcomes, or complications. Screening, data extraction, and quality assessment using the 13-item JBI Critical Appraisal Checklist for RCTs were performed independently by two reviewers. Findings were synthesised descriptively using a best-evidence approach.

**Results:**

Of 390 unique records, five RCTs were included. Studies investigated lidocaine in various formulations and administration routes, including spray, drops, gel, and atomised lidocaine, compared with placebo. Despite heterogeneity in study characteristics, all studies reported reductions in pain or discomfort scores with lidocaine. Best-evidence synthesis showed strong evidence for pain reduction and moderate evidence for reductions in discomfort and cough. Evidence for other procedure-related outcomes was insufficient.

**Conclusion:**

Topical lidocaine during NGT insertion in adults appears to reduce pain and is well tolerated. However, heterogeneity and limited data preclude strong recommendations. Further high-quality trials are warranted to identify optimal formulation, dosage, and administration route for routine practice.

**Registration:**

The review was registered in the international prospective register of systematic reviews (http://www.crd.york.ac.uk/PROSPERO/), registration number: CRD420251177888.

**What is already known**

• - Nasogastric tube insertion is a common hospital procedure that is often associated with substantial pain and discomfort.
• - Previous studies have shown that lidocaine may reduce pain during nasogastric tube insertion.
• - The optimal formulation, dosage, and administration route of lidocaine for routine clinical practice remain unclear.

**What this paper adds**

• - This systematic review found strong evidence that topical lidocaine reduces pain during nasogastric tube insertion in adults.
• - Moderate evidence suggests that lidocaine also reduces procedural discomfort and cough.
• - Current evidence is insufficient to determine the most effective and clinically feasible lidocaine formulation, dosage, or administration route.

## 1 Introduction

Nasogastric tubes (NGTs) insertion is a common procedure in hospitals and has been described as one of the most painful routine procedures.(Singer and Konia, 1999) A NGT is a flexible tube inserted through the nasal passage into the stomach, and is used for administration of nutrition, fluids, or medication, stomach lavage, and gastric drainage.(Thomsen et al., 2006) Pain and discomfort during NGT insertion are mainly caused by mechanical trauma to the nasal mucosa. In a 2005 survey, over 90% of emergency department physicians acknowledged the painful nature of the procedure and reported strategies to mitigate discomfort, yet fewer than 30% considered the available methods sufficiently effective.(Juhl and Conners, 2005) In practice, NGT insertion is often performed with no gel or with lubricating gel only, without additional anaesthetic agents. Barriers to optimal pain management include lack of standardized protocols or guidelines, lack of acknowledgement of patients’ discomfort, insufficient clinician knowledge, and limited availability or perceived inconvenience of alternative options.(Czarnecki et al., 2011, Solomon and Jurica, 2017)

Several randomized controlled trials (RCTs) and cross-over studies have evaluated the efficacy of lidocaine in reducing pain during NGT insertion, demonstrating improved patient comfort across formulations such as gel, spray, nebulizer, atomizer, and drops.(Babl et al., 2009, Ducharme and Matheson, 2003, Singer and Konia, 1999, Wolfe et al., 2000) Two systematic literature reviews, synthesizing RCTs up to 2017 concluded that lidocaine effectively reduces procedural pain and discomfort,(Lor et al., 2018, Pinto et al., 2020) without increasing complication rates.(Lor et al., 2018) However, these reviews did not distinguish between formulations in terms of clinical feasibility or availability in routine care. This is clinically relevant, as some formulations require dedicated delivery devices or additional preparation, which may limit feasibility for routine bedside use. The Dutch guideline on NGT placement recommends lidocaine only for inserting larger-diameter tubes in the emergency department, without specifying optimal dosage, formulation, or administration route.(V&VN, 2017)

Since previous reviews included studies only up to 2017, an updated synthesis is warranted to capture new evidence. In addition, previous reviews did not consider the feasibility and availability of interventions in routine clinical settings, which may hamper real-world implementation in hospitals. Therefore, this study aims to perform an updated systematic review and best evidence synthesis on the effectiveness and potential adverse effects of local anaesthetic and/or lubricant agents in reducing pain and discomfort during NGT insertion in adults, with particular emphasis on interventions that are safe to use, practical and readily available in routine hospital care, defined as agents that can be administered at the bedside without additional equipment or alteration of the level of consciousness.

## 2 Method

The Preferred Reporting Items for Systematic Reviews and Meta-Analyses was used to report this review.(Page et al., 2021) The review was registered in the international prospective register of systematic reviews (http://www.crd.york.ac.uk/PROSPERO/), registration number: CRD420251177888.

### 2.1 Search strategy

We conducted a systematic search from database inception to October 28^th^, 2025 to identify studies examining pain relief during NGT insertion. The literature search included MEDLINE (via PubMed), Cochrane Database of Systematic Reviews and CENTRAL (both via Cochrane library), and CINAHL (via EBSCO). The search strategy was structured using a PICO framework. We combined terms related to ‘nasogastric tube’, ‘insertion’, ‘topical anaesthetics’, and ‘lidocaine’ using Boolean operators, without applying restrictions on date, language or other factors. To support and refine the search, ChatGPT (OpenAI) was used to suggest synonyms and adapt the PubMed query for the other databases, after which a researcher manually reviewed and verified all suggestions.

The search strategy was validated by confirming that it retrieved key articles identified in previous systematic reviews,(Uri et al., 2011, Wolfe et al., 2000) after which minor adjustments were made before the final search run. Reference lists of included articles and relevant systematic reviews were screened to identify additionally eligible studies. The full search strategy is provided in Table 1.

**Table 1:** Search strategy.

**MEDLINE via Pubmed**
"Intubation, Gastrointestinal"[Mesh] OR ((Nasogastric[tiab] OR gastrointestinal[tiab] OR NG[tiab] OR nasal[tiab] OR NGT[tiab]) AND (Insertion*[tiab] OR "Intubation"[Mesh] OR intubation*[tiab] OR placement*[tiab])) AND "Lubricants"[Mesh] OR lubricant*[tiab] OR lubricating[tiab] OR lubrication[tiab] OR "Anesthetics, Local"[Mesh] OR ((Topical[tiab] OR local[tiab]) AND (anesthetic*[tiab] OR "Anesthetics"[Mesh])) OR "Lidocaine"[Mesh] OR lidocaine[tiab] OR lignocaine[tiab] OR Xylocaine[tiab] OR instillagel[tiab]
**Cochrane Database of Systematic Reviews (CDSR) and CENTRAL via Cochrane library**
[mh "Intubation, Gastrointestinal"] OR ((nasogastric OR gastrointestinal OR NG OR nasal OR NGT):ti,ab AND ([mh Intubation] OR (insertion* OR intubation* OR placement*):ti,ab)) AND [mh Lubricants] OR (lubricant* OR lubricating OR lubrication):ti,ab OR ((topical OR local):ti,ab AND (anesthetic*:ti,ab OR [mh Anesthesia])) OR [mh Lidocaine] OR (lidocaine OR lignocaine OR Xylocaine OR instillagel):ti,ab
**CINAHL (via EBSCO)**
(MH "Intubation, Gastrointestinal" OR MH "Gastrointestinal Intubation (Iowa NIC)") OR ((TI nasogastric OR AB nasogastric OR TI gastrointestinal OR AB gastrointestinal OR TI NG OR AB NG OR TI nasal OR AB nasal OR TI NGT OR AB NGT) AND (TI insertion* OR AB insertion* OR MH "Intubation+" OR TI intubation* OR AB intubation* OR TI placement* OR AB placement*)) AND (MH "Lubricants") OR TI lubricant* OR AB lubricant* OR TI lubricating OR AB lubricating OR TI lubrication OR AB lubrication OR (MH "Anesthetics, Local+") OR (MH "Anesthesia, Local") OR ((TI topical OR AB topical OR TI local OR AB local) AND (TI anesthetic* OR AB anesthetic* OR MH "Anesthetics+" OR MH "Anesthesia+")) OR (MH "Lidocaine") OR TI lidocaine OR AB lidocaine OR TI lignocaine OR AB lignocaine OR TI Xylocaine OR AB Xylocaine OR TI instillagel OR AB instillagel

### 2.2 Eligibility criteria

Studies were included if they exclusively involved adults (≥18 years of age) undergoing NGT insertion and investigated any intervention to reduce pain during NGT insertion, such as lubricants or topical anaesthetics (e.g., lidocaine) administered via a non-nebulised route. Comparators could include placebo, no intervention, or an alternative administration route of the same agent (excluding nebulised forms). Eligible outcomes included pain or discomfort during or immediately after NGT insertion, procedure-related outcomes (e.g., number of attempts) or procedural complications. Only RCTs, including crossover design, published in English, German, French or Dutch were included.

Studies were excluded if they evaluated medical procedures other than nasogastric tube insertion (e.g., tracheal intubation, endoscopy), involved sedatives or consciousness-altering agents (e.g., midazolam, ketamine), combined lidocaine with other anaesthetics, or used nebulised agents. Studies reporting only physiological outcomes (e.g., heart rate, blood pressure), lacking statistical analysis, or published in non-peer-reviewed sources (e.g., letters to the editor, conference abstracts, commentaries), as well as systematic or narrative reviews, were excluded from data extraction.

### 2.3 Selection process

All identified records were imported into Rayyan.ai. Automatically identified duplicates were manually reviewed and removed. First, the titles and abstracts were screened, followed by full-texts screening of potentially eligible articles. Two reviewers (FvK, KK) performed both screening phases independently and in duplicate. Discrepancies were resolved through discussion.

### 2.4 Data extraction

A standardised data extraction form was developed and piloted with one study. Once finalised, one reviewer (FvK) extracted the data, which was crosschecked by a second reviewer (KK). Extracted information included: study characteristics (i.e., country, design, setting, study period, sample size, study population characteristics), intervention and control details (i.e., drug type, formulation, administration route, dosage, standard procedure), procedural details (i.e., tube size, timing, operator), outcomes, and reported study limitations.

### 2.5 Critical appraisal

The methodological quality of included studies was independently assessed by two reviewers (FvK, KK) using the 13-item JBI Critical Appraisal Checklist for Randomised Controlled Trials.(Barker et al., 2024) The checklist assessed the following aspects: randomisation, allocation concealment, baseline comparability, blinding, outcome measurement, follow-up, and statistical analysis. Discrepancies were resolved through discussion until consensus was reached. An overall level of quality was given to each individual study based on the number of criteria met: low quality (0-4), medium quality (5-8) and high quality (>8).(Fernandez et al., 2021) All studies were included in the review regardless of quality rating.

### 2.6 Data analysis

Given the anticipated clinical and methodological heterogeneity in both interventions and outcome measures, a meta-analysis was not planned a priori. Instead, a qualitative best evidence synthesis was conducted for each outcome, taking into account both the number of studies and their methodological quality (Slavin, 1995, Van Tulder et al., 2003, Veronese et al., 2016). Study characteristics and findings were summarised descriptively, with data grouped per outcome. The certainty of evidence was therefore not formally assessed using the GRADE approach.

The strength of evidence was classified as: (1) strong evidence: consistent findings across at least two high-quality studies; (2) moderate evidence: consistent findings in one high-quality study supported by at least one moderate- or low-quality study, or consistent results across multiple moderate- or low-quality studies; (3) insufficient evidence: fewer than two studies available, or inconsistent findings across studies. Findings were considered consistent when at least 75% of studies reported results in the same direction.

### 2.7 Ethical approval

Ethical approval was not required for this study, as it is a systematic review of previously published data.

## 3 Results

From the initial 859 records, 390 unique titles and abstracts were screened after removal of duplicates. Thirty-six articles were selected for full-text review. Nine full-texts could not be retrieved, all were trial references identified in Cochrane without associated publications. After full-text assessment, 22 articles were excluded (Figure 1), resulting in five studies eligible for inclusion. Reference list screening did not identify additional eligible studies beyond those retrieved through the database search. The most common primary reasons for exclusion were non-relevant publication type (n = 8),(Edwards, 1993, Fowler, 2011, Gallagher, 2004, Kuo et al., 2010, Lor et al., 2018, Schmidt, 2005, West, 1982, Wyer, 2005) interventions outside the scope of this review (n = 6),(Cullen et al., 2004, Manning et al., 2016, Nejati et al., 2010, Nott and Hughes, 1995, Rouhi et al., 2020, Singer and Konia, 1999) and studies conducted in children (n = 3).(Babl et al., 2009, Craig et al., 2019, Farrington et al., 2015)

**Fig. 1:**
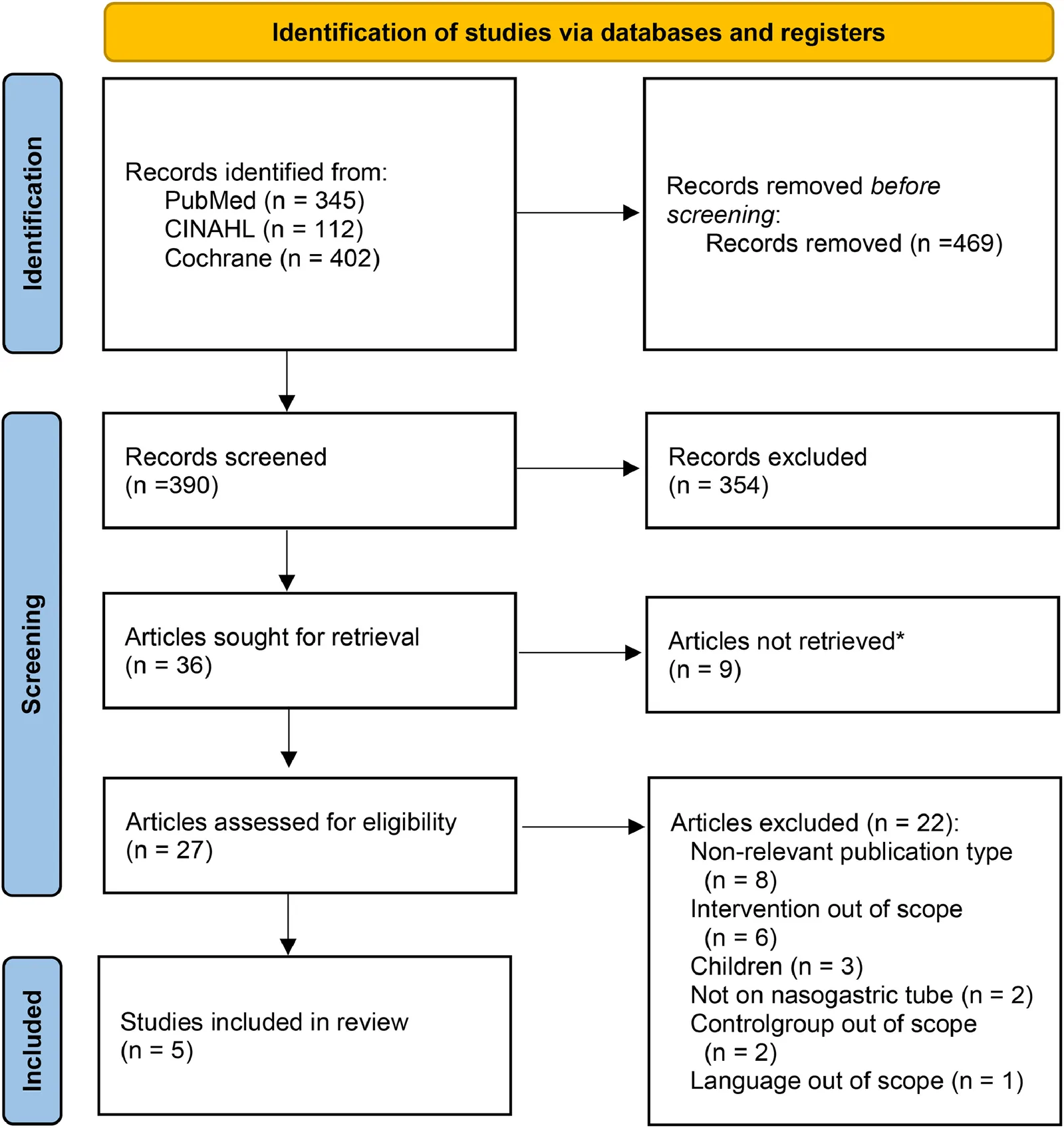
PRISMA flowchart illustrating the identification, screening, eligibility assessment, and inclusion of studies in this systematic review. * The records not retrieved were references to trials found in Cochrane without associated publications.

### 3.1 Study characteristics

The five included studies were all double-blinded RCTs enrolling hospitalised adult patients, predominantly in emergency settings (see Table 2 for study characteristics). Sample sizes ranged from 40 to 206 participants. Four studies were conducted in Asia(Chan and Lau, 2010, Pongprasobchai et al., 2007, Tongyoo et al., 2024, Uri et al., 2011) and one in North America.(Wolfe et al., 2000) Reported study periods spanned 2005 to 2022, while one earlier study published in 2000 did not specify its study period.

**Table 2:** Study characteristics table.

Ref Country Study design	Study population Study period	No of patients	Intervention	Description	Reported outcomes	Methodological quality⁎
			Control			
Chan, 2010 Hong Kong RCT	Adults in emergency department May – October 2005	206 I: 103 C: 103	10% lidocaine spray	- 1 spray of 1mL in both nostrils and 2 sprays of 1mL in throat - Standard procedure: 1 ml KY jelly lubrication - 5 min before 16F intubation	Discomfort; Difficulty insertion; Insertion time; No. of attempts; Failed insertions; Vital signs; adverse events	Moderate quality
			Placebo: saline spray			
Tongyoo, 2024 Thailand RCT	Adults in emergency department September 2021 – August 2022	126 I: 63# C: 63⁎	2% lidocaine drops	- 2 to 3 drops (0.2-0.3mL) in one nostril - Standard procedure: lubricant jelly without lidocaine - 2 min before 12F intubation	Pain; Insertion time; No. of attempts; adverse events	Moderate quality
			Placebo: saline drops			
Pongprasobchai, 2007 Thailand RCT	Adult inpatient and outpatient in Department of Internal Medicine October – December 2006	60 I: 31 C: 29	10% lidocaine spray	- 2 puffs in each nostril & 6 puffs in throat (total 1.4mL) - Standard procedure: 3mL of 2% lidocaine jelly - 3 min before 14F or 18F intubation	Pain; Discomfort; Satisfaction with intervention before intubation (patient & physician); Same spray selection next intubation (patient & physician); Difficulty insertion; Insertion time; No. of attempts; Failure rate; adverse events	Moderate quality
			Placebo: saline spray			
Uri, 2011 Israel RCT	Convenience sample of adults in emergency department October 2007 - March 2008	62 I: 31 C: 31	2% lidocaine gel	- 5 mL gel instilled in nostril, inhaled and swallowed - Standard care: tip of tube lubricated with small amount of placebo gel + sip of water during insertion - 5 min before 16F intubation	Pain (overall & nasal); Difficulty insertion; No. of attempts, adverse events	High quality
			Placebo: water-based lubricant gel			
Wolfe, 2000 United Stated of America RCT	Convenience sample of adults in emergency department Study period not reported	40 I: 20 C: 20	4% lidocaine atomised	- 1.5 mL atomized in nasopharynx, 3 mL atomized in oropharynx - Standard procedure: 5mL of 2% lidocaine jelly intranasal injected - 18F intubation directly immediately after application	Pain; Unsuccessful NGT placements	High quality
			Placebo: saline solution atomised			

C, control; I, intervention; min, minute(s); mL, millilitre; No, number; NGT, nasogastric tube; RCT, randomised controlled trial; .

⁎ For elaborated methodological quality assessment, see Table 3

# Group sizes were not explicitly reported but approximate numbers could be inferred from a figure.

All included studies investigated lidocaine as the intervention, with variation in both formulation and administration route. Two studies assessed 10% lidocaine spray applied to both nostrils and the throat.(Chan and Lau, 2010, Pongprasobchai et al., 2007) Other interventions consisted of 2% lidocaine drops applied to one nostril,(Tongyoo et al., 2024) 2% lidocaine gel instilled intranasally and swallowed,(Uri et al., 2011) and 4% lidocaine atomised into the nasopharynx and oropharynx.(Wolfe et al., 2000) All studies used placebo comparators that mimicked the intervention in appearance and administration route. Standard care during NGT insertion consisted of lubrication jelly, administered either intranasally or directly to the tube. The interval between intervention and tube insertion ranged from immediate insertion(Wolfe et al., 2000) to up to five minutes.(Chan and Lau, 2010, Uri et al., 2011) Nasogastric tube size varied between 12F(Tongyoo et al., 2024) and 18F.(Wolfe et al., 2000, Pongprasobchai et al., 2007)

All studies measured pain or discomfort related to the procedure directly after the insertion, using a visual analogue scale (VAS). Additional outcomes included difficulty of insertion,(Chan and Lau, 2010, Pongprasobchai et al., 2007, Uri et al., 2011) insertion time,(Chan and Lau, 2010, Pongprasobchai et al., 2007, Tongyoo et al., 2024) number of attempts,(Chan and Lau, 2010, Pongprasobchai et al., 2007, Tongyoo et al., 2024, Uri et al., 2011, Wolfe et al., 2000) and adverse events.(Chan and Lau, 2010, Pongprasobchai et al., 2007, Tongyoo et al., 2024, Uri et al., 2011)

### 3.2 Critical appraisal

Methodological quality assessments are summarised in Table 3. Three studies were rated as moderate quality, mainly due to limitations in randomisation, allocation concealment, blinding, and statistical analysis.(Chan and Lau, 2010, Pongprasobchai et al., 2007, Tongyoo et al., 2024) The remaining two studies were rated as high methodological quality.(Uri et al., 2011, Wolfe et al., 2000)

**Table 3:** Methodological quality assessment of the included studies.

Reference	Chan, 2010	Tongyoo, 2024	Pongprasobchai, 2007	Uri, 2011	Wolfe, 2000
**Was true randomization used for assignment of participants to treatment groups?**	Yes	No	Unclear	Yes	Yes
**Was allocation to treatment groups concealed?**	Yes	Unclear	Unclear	Yes	Yes
**Were treatment groups similar at the baseline?**	Yes	No	No	Yes	Unclear
**Were participants blind to treatment assignment?**⁎	Unclear	Yes	Unclear	Yes	Yes
**Were those delivering treatment blind to treatment assignment?**⁎	Unclear	Unclear	Yes	Yes	Yes
**Were outcomes assessors blind to treatment assignment?**	No	Yes	Yes	Yes	Yes
**Were treatment groups treated identically other than the intervention of interest?**	Yes	Yes	Yes	Yes	Yes
**Was follow up complete and if not, were differences between groups in terms of their follow up adequately described and analyzed?**	Yes	No	Unclear	Unclear	Yes
**Were participants analyzed in the groups to which they were randomized?**	No	Unclear	Yes	Unclear	Yes
**Were outcomes measured in the same way for treatment groups?**	Yes	Yes	Yes	Yes	Yes
**Were outcomes measured in a reliable way?**	Unclear	Unclear	Yes	Yes	Yes
**Was appropriate statistical analysis used?**	No	No	Yes	No	No
**Was the trial design appropriate, and any deviations from the standard RCT design (individual randomization, parallel groups) accounted for in the conduct and analysis of the trial?**	Yes	Yes	Yes	Yes	No
**RISK OF BIAS**	Moderate risk of bias	Moderate risk of bias	Moderate risk of bias	Low risk of bias	Low risk of bias
**OVERALL APPRAISAL**	**Moderate methodological quality**	**Moderate methodological quality**	**Moderate methodological quality**	**High methodological quality**	**High methodological quality**

⁎ Although all studies were described as double-blind, the adequacy of blinding was sometimes judged as unclear, as reported differences in smell and taste between lidocaine and placebo may have allowed patients and staff to identify the intervention.

### 3.3 Pain and discomfort related to NGT insertion

**Pain (VAS-100mm)**. Four studies assessed the effect of lidocaine on pain during NGT insertion.(Pongprasobchai et al., 2007, Tongyoo et al., 2024, Uri et al., 2011, Wolfe et al., 2000) All reported significantly lower mean pain scores in the intervention groups compared with placebo, with absolute differences ranging from 14 mm to 31 mm (Table 4).

**Table 4:** Pain, discomfort, and procedure-related outcomes.

*Reference*	Formulation	Intervention group	Absolute difference± (95% CI)	p-value	Favours
		Control group			
**Pain and discomfort**					
**Pain score in VAS-100mm, mean (SD) (95% CI)**					
*Tongyoo, 2024*	Drops	14.1 (5.0)$	31.3#	p<0.01	**Intervention**
		45.4 (10.3)$			
*Pongprasobchai, 2007*	Spray	23.6 (16.6)	19.6 (6.7 to 32.4)	0.007≠	**Intervention**
		43.1 (31.4)			
*Uri* *(overal pain)*	Gel	37 (28)	14 (11 to 17)	<0.05	**Intervention**
		51 (26)			
*Uri, 2011* *(nasal pain)*	Gel	33 (29)	15 (12 to 18)	0.05	**Intervention**
		48 (27)			
*Wolfe, 2000*	Atomised	37.4 (25.6)£	27.1#	n.r.⁎	**Intervention**
		64.5 (27.0)£			
**Discomfort score in VAS-100mm, mean (SD)**					
*Chan, 2010*	Spray	20 (n.r.)$	70# (8-16)¥	<0.005	**Intervention**
		90 (n.r.)$			
*Pongprasobchai, 2007*≠	Spray	30.0 (24.4)	21.4 (7.3 to 35.5)	0.009≠	**Intervention**
		51.4 (30.0)			

**Procedure-related outcomes**					
**Difficulty insertion in 5-point likert scale, mean (SD)/median (IQR)**					
*Chan, 2010*	Spray	Mean: 1 (n.r.)	2# (4-12)¥	<0.005	**Intervention**
		Mean: 3 (n.r.)			
*Uri, 2011*	Gel	Mean: 2.1 (0.9)	0.7 (0.25 to 1.15)	<0.05	**Control**
		Mean: 1.4 (0.7)			
*Pongprasobchai, 2007*	Spray	Median: 2 (1-3)	1#	0.255	**-**
		Median: 3 (2-3)			
**Insertion time, mean (SD)**					
*Tongyoo, 2024*	Drops	1.52 minutes (0.76)	1.86#	<0.01	**Intervention**
		3.38 minutes (1.36)			
*Chan, 2010*	Spray	1.5 minutes (n.r.)	3# (7-14)¥	<0.005	**Intervention**
		4.5 minutes (n.r.)			
*Pongprasobchai, 2007*	Spray	65.3 seconds (34.1)	28.4 seconds (-8.6 to 65.5)	0.689	-
		93.8 seconds (96.8)			
**Number of attempts, mean (SD) / %**					
*Chan, 2010*	Spray	Mean: 1 (n.r.)	1# (8-15)¥	<0.005	**Intervention**
		Mean: 2 (n.r.)			
*Chan, 2010*	Spray	≥2 attempts: 10 %	75# (3-15)¥	<0.05	**Intervention**
		≥2 attempts: 85%			
*Tongyoo, 2024*	Drops	1: 98.4% 2: 0.0% 3: 1.6% 4: 0.0%	-	<0.01	**Intervention**
		1: 0.0% 2: 50.8% 3: 47.6% 4: 1.6%			
*Pongprasobchai, 2007*	Spray	1: 80.6% ≥2: 19.4%	-	0.653	**-**
		1: 79.3% ≥2: 20.7%			
*Pongprasobchai, 2007*	Spray	>3 attempts: 0.0%	6.9#	0.229	**-**
		>3 attempts: 6.9%			
*Uri, 2011*	Gel	1.4 (0.7)	0.2 (-0.2 to 0.6)	n.s.	**-**
		1.2 (0.6)			
*Wolfe, 2000*	Atomised	1: 100%	-	-	-
		One unsuccesful NGT placement; futher attempts refused			

CI, confidence interval; IQR, interquartile range; mm, milimeter; NGT, nasogastric tube; n.r., not reported; n.s., not significant; SD, standard deviation; VAS, visual analogue scale.

± For consistency, all differences are presented as absolute values;

$ VAS-10 converted to VAS-100 for comparability;

£ SDs were not reported and therefor calculated by the authors from the sample size (n=20 in each group) and the reported 95% CI (intervention group: 25.4-49.4; control group: 51.8-77.1);

⁎ in tekst mentioned that it was statistically significant but p-value not reported;

# Absolute difference calculated by authors;

≠ After correcting for patient’s gender, types of treatment were still significantly associated with degree of pain (p=0.007) and discomfort (p=0.009).

¥ Adjusted mean difference estimated using ANCOVA; not raw difference between group means;

**Discomfort (VAS-100mm)**. Two studies evaluated discomfort.(Chan and Lau, 2010, Pongprasobchai et al., 2007) Consistent with the findings for pain, both reported significantly lower mean discomfort scores in the lidocaine groups (20mm – 30mm) compared with placebo (51mm – 90mm), corresponding to absolute differences of 21 mm and 70 mm.

### 3.4 Procedure-related outcomes

**Difficulty of insertion (5-point Likert scale)**. Three studies assessed the perceived difficulty of NGT insertion according to the healthcare professional.(Chan and Lau, 2010, Pongprasobchai et al., 2007, Uri et al., 2011) One study reported significantly lower mean difficulty scores with lidocaine spray compared to placebo (1 vs. 3),(Chan and Lau, 2010) while another study found significantly higher mean difficulty scores with lidocaine gel (2.1 vs. 1.4).(Uri et al., 2011) The third study reported no significant difference on difficulty of insertion between the intervention and control group.(Pongprasobchai et al., 2007)

**Insertion time (minutes)**. Three studies evaluated insertion time.(Chan and Lau, 2010, Pongprasobchai et al., 2007, Tongyoo et al., 2024) In two, insertion time was significantly shorter in the lidocaine groups (1.5 vs. 3.4 minutes(Tongyoo et al., 2024); and 1.5 vs. 4.5 minutes(Chan and Lau, 2010)), while one study reported no significant difference.(Pongprasobchai et al., 2007)

**Number of attempts (n)**. All five studies reported on the number of attempts to insert the NGT. In two studies, no significant difference was observed between the intervention and control groups.(Uri et al., 2011, Pongprasobchai et al., 2007) In contrast, Chan and Lau (2010) reported a higher number of attempts in the control group compared with the intervention group (mean: 2 vs. 1; ≥2 attempts: 85% vs. 10%). Similarly, Tongyoo et al. (2024) found that almost all patients in the intervention group required a single attempt (98.4%) whereas the control group more often required two (50.8%) or three attempts (47.6%). Wolfe et al. (2000) reported one unsuccessful NGT placement but no statistical testing was provided.

### 3.5 Adverse events

The most frequently reported adverse events were difficulty breathing, epistaxis, vomiting, and cough, see Table 5.(Chan and Lau, 2010, Pongprasobchai et al., 2007, Tongyoo et al., 2024, Uri et al., 2011)

**Table 5:** Adverse events related to NGT insertion$.

**Reference**	**Formulation**	**Intervention**	**Absolute difference**±**(95% CI)**	**p-value**	**Favours**
		**Control**			
**Adverse events**					
**Difficulty of breathing**$**in frequency (%)/ VAS-100mm (mean (SD))**					
*Chan, 2010*	Spray	1%	40%# (5% to 10%)¥	<0.001⁎	**Intervention**
		41%			
*Tongyoo, 2024*	Drops	0.0%	15.9#	<0.01	**Intervention**
		15.9%			
*Pongprasobchai, 2007*	Spray	0.0%	0.0#	-	**-**
		0.0%			
*Uri, 2011*	Gel	VAS-100: 27 (26)	15 (12 to 17)	n.s.	**-**
		VAS-100: 42 (32)			
**Epistaxis in frequency (%)**					
*Chan, 2010*	Spray	2%	10%# (7% to 16%)¥	0.017⁎	**Intervention**
		12%			
*Tongyoo, 2024*	Drops	17.5%	1.6#	0.81	**-**
		15.9%			
*Pongprasobchai, 2007*	Spray	0.0%	0.0#	-	**-**
		0.0%			
**Vomit in frequency (%)**					
Chan, 2010	Spray	4%	61%# (4% to 9%)¥	<0.001⁎	**Intervention**
		65%			
*Tongyoo, 2024*	Drops	0.0%	30.2#	<0.01	**Intervention**
		30.2%			
*Uri, 2011*	Gel	9.7%	0 (-16% to 16%)	-	**-**
		9.7%			
**Cough in frequency (%)**					
Chan, 2010	Spray	8%	11%# (3% to 8%)¥	0.015⁎	**intervention**
		19%			
*Tongyoo, 2024*	Drops	0.0%	19.0#	<0.01	**Intervention**
		19.0%			
**Chest pain in frequency (%)**					
Chan, 2010	Spray	0%	12%# (3% to 18%)¥	0.002⁎	**Intervention**
		12%			
**Nausea in VAS-100mm, mean (SD)**					
*Uri, 2011*	Gel	25 (29)	15 (12 to 18)	n.s.	**-**
		40 (38)			
**Dizziness in frequency (%)**					
Chan, 2010	Spray	0%	12%# (6% to 14%)¥	0.002⁎	**Intervention**
		12%			
**Epigastric pain in frequency (%)**					
Chan, 2010	Spray	0%	10%# (7% to 13%)¥	0.001⁎	**Intervention**
		10%			
**Face petechiae in frequency (%)**					
Chan, 2010	Spray	0%	6%# (2% to 18%)¥	0.013⁎	**Intervention**
		6%			
**Palpitation in frequency (%)**					
Chan, 2010	Spray	1%	3%# (-2% to 9%)¥	0.36⁎	**-**
		4%			
**Gagging sensation in VAS-100mm (mean (SD))**					
*Uri, 2011*	Gel	25 (30)	14 (11 to 17)	<0.05	**Intervention**
		39 (24)			

**Procedure-related complication**					
**Aspiration in frequency (%)**					
*Tongyoo, 2024*	Drops	0%	6.3#	0.04	**Intervention**
		6.3%			
**Tracheal insertion in frequency (%)**					
*Pongprasobchai, 2007*	Spray	0.0%	0.0#	-	**-**
		0.0%			

CI, confidence interval; mm, milimeter; NGT, nasogastric tube; n.s., not significant; SD, standard deviation; VAS, visual analogue scale.

± For consistency, all differences are presented as absolute values;

$ In Chan (2010), adverse event oucomes Numbness, Allergy, and Tracheal insert is reported as 0% vs 0% with a p<0.001. This is likely a reporting error. We extracted the event rates as 0% vs 0%, and considered the effect as neutral. The reported p-value and CI were disregarded;

⁎ analysis of covariance

# Difference calculated by authors;

$ Defined as ”Shortness of breath” (Chan, 2010), ”difficulty breathing” (Tongyoo, 2024), ”choking” (Pongprasobchai, 2007), ”chocking sensation” (Uri, 2011);

¥ Adjusted mean difference estimated using ANCOVA; not raw difference between group means.

For difficulty breathing, four studies reported mixed findings: two found lower rates with lidocaine compared to the control group (0–1% vs 16–41,(Chan and Lau, 2010, Tongyoo et al., 2024) one reported no difference (VAS-100: 27mm vs 42mm)(Uri et al., 2011), and one observed no events in either group.(Pongprasobchai et al., 2007) Three studies reported on epistaxis with inconsistent findings: fewer events in the intervention group (2% vs 12%),(Chan and Lau, 2010) no difference between the groups (18% vs 16%),(Tongyoo et al., 2024) and one reported no events in either group.(Pongprasobchai et al., 2007) Vomiting was assessed in three studies: two reported lower rates with lidocaine (0–4% vs 30–65),(Chan and Lau, 2010, Tongyoo et al., 2024) while one found the same percentages in either group (9.7%).(Pongprasobchai et al., 2007) Cough was reported in two studies, both showing fewer events in the intervention group (0–8% vs 19%).(Chan and Lau, 2010, Tongyoo et al., 2024)

Several additional adverse events were reported in individual studies. In the study byChan and Lau (2010), rates of chest pain, dizziness, epigastric pain, and face petechiae were lower in the intervention group in the study, whereas the rate of palpitations did not differ between the groups. Nausea was similar between groups, though gagging sensation was reduced with lidocaine.(Uri et al., 2011) Aspiration was less frequent in the intervention group,(Tongyoo et al., 2024) and no tracheal insertions were observed in either group.(Pongprasobchai et al., 2007)

### 3.6 Strength of evidence

Using the definitions for the best-evidence synthesis described in the Methods section, there is strong evidence that lidocaine reduces pain during NGT insertion. For discomfort and cough reduction, the studies provide moderate evidence for an effect of lidocaine. The evidence for all other procedure-related outcomes, including difficulty of insertion, insertion time, number of attempts, and adverse events other than cough, was insufficient to support an effect of lidocaine.

## 4 Discussion

This systematic review evaluated the effectiveness and safety of topical anaesthetics and lubricants for reducing pain and discomfort during nasogastric tube insertion in adults. The review found strong evidence that lidocaine reduces pain, moderate evidence for reductions in discomfort and cough, and insufficient evidence for other procedure-related outcomes. Studies varied in formulation, dosage, administration route, and timing, resulting in substantial heterogeneity.

Since the last systematic review by Pinto et al. (2020), only one new study has emerged, which we have included in the present review.(Tongyoo et al., 2024) This highlights that relatively little research has been conducted on this common procedure. Consequently, it remains unclear which formulation, route, or timing of administration is most effective and practical, and it is remarkable that such a common procedure has not been investigated more thoroughly. Most studies were conducted in Asia and are relatively old, emphasizing the need for further research in European populations to confirm generalizability and to determine the most effective and practical approach for routine practice. Our findings are consistent with previous systematic reviews, demonstrating that lidocaine reduces pain associated with nasogastric tube insertion.(Kuo et al., 2010, Lor et al., 2018, Pinto et al., 2020) The magnitude of pain reduction observed in our review supports the clinical relevance of the intervention: all studies exceeded the 13 mm threshold for minimum clinically significant change on a 100-mm VAS,(Todd et al., 1996) with reductions ranging from 15 mm (Uri et al., 2011) to 31 mm.(Tongyoo et al., 2024) These findings indicate that lidocaine interventions provide not only statistically significant but also clinically meaningful relief of pain. Remarkably, in three of the five included studies, the procedure was performed before lidocaine reached its maximal effect, suggesting that the observed pain reduction might have been even greater if administration had been optimally timed. Furthermore, one study specifically compared lidocaine gel, administered intranasally and swallowed, with standard gel without lidocaine, finding that the intervention group experienced less pain and discomfort.(Uri et al., 2011) This result highlights the added value of lidocaine when incorporated into the gel formulation. This review focused on adults to ensure consistent outcome assessment, as pain and discomfort are measured differently in children. Nevertheless, the findings might be relevant for future paediatric practice.

Regarding safety, previous reviews rarely addressed adverse events; only Lor et al. (2018) briefly discussed nasal bleeding and cough, reporting no serious complications. Our review systematically extracted and summarized safety outcomes, showing that lidocaine was generally well tolerated, with adverse events infrequent and mostly comparable between groups. These findings support the use of lidocaine as a safe option for pain management during nasogastric tube insertion, even though the optimal dosage, formulation, and timing have not yet been established.

Finally, Uri et al. (2011) explored demographic and clinical predictors of pain scores. In multivariable analysis, the difficulty of the procedure emerged as the only independent predictor of pain; sex, age, and prior experience were not found to be significant. Similarly, in another study, although the lidocaine group included more female patients, multivariate analysis showed that after correction for gender, only the treatment type (lidocaine vs. placebo) remained significantly associated with pain and discomfort. This suggests that, for nasogastric tube insertion, pain is largely influenced by procedural factors and the skill or technique of the healthcare professional, rather than patient characteristics, highlighting the importance of operator training and careful technique in addition to pharmacological pain management. Another possibility is that the interaction and collaboration between the healthcare professional and the patient influence the level of comfort during the procedure. However, these findings are based on single studies and should be confirmed in further research.

This review was reported in accordance with the Preferred Reporting Items for Systematic Reviews and Meta-Analyses 2020 guidelines. A comprehensive search across multiple databases was performed, with independent screening and data extraction conducted by two reviewers, and formal quality assessment of included studies. The absence of additional studies identified through reference list screening suggested that the database search strategy was comprehensive. Only randomized controlled trials of moderate to highmethodological quality were included, ensuring both a high level of evidence and methodological reliability. Beyond these essential standards, the review focused specifically on adult patients undergoing nasogastric tube insertion and evaluated only non-nebulised topical anaesthetics or lubricants, ensuring the clinical relevance of the findings. The review captured outcomes important to patients, such as pain, discomfort, and cough, alongside procedure-related outcomes, enhancing the practical applicability of the results.

Several limitations should be acknowledged. Although this review aimed to assess a range of topical anaesthetics and lubricants, all included studies evaluated lidocaine, reflecting its predominant use in clinical practice. The relatively small number of included studies limits the generalizability of the findings. In addition, substantial heterogeneity in lidocaine formulations, dosages, administration routes, tube sizes, and timing complicated direct comparisons across studies and precluded meta-analysis. Although overall quality was moderate to high, statistical analyses and reporting were not optimal in some studies. Finally, evidence on procedure-related outcomes beyond pain, such as insertion difficulty, number of attempts, and adverse events, was limited.

Despite evidence supporting lidocaine for pain reduction, the heterogeneity of formulations, dosages, administration routes, and study designs limits clear recommendations for clinical practice. Future research should focus on adequately powered, high-quality randomized controlled trials that directly compare different lidocaine formulations and administration methods. Studies should also assess procedure-related outcomes such as insertion difficulty, number of attempts, procedure duration, and adverse events, alongside patient-centered outcomes like discomfort and cough. Evaluating feasibility and acceptability in routine hospital settings will be essential to develop standardized, evidence-based protocols for nasogastric tube insertion using lidocaine in adults.

## 5 Conclusion

This systematic review indicates that topical lidocaine during nasogastric tube insertion in hospitalised adults reduces pain and it is generally well tolerated, with moderate evidence for reductions in discomfort and cough. However, evidence for other procedure-related outcomes including insertion difficulty, number of attempts, procedure duration, and adverse events other than cough is insufficient. The included studies varied in formulation, administration route, and timing. Methodological quality ranged from moderate to high, highlighting heterogeneity in the available evidence. Due to these variations and the limited number of studies, firm recommendations regarding the optimal formulation, dosage, and administration route for routine clinical practice cannot yet be made. Future research should focus on adequately powered, high-quality RCTs that compare different lidocaine formulations and administration routes, evaluate both patient-centered outcomes and procedure-related outcomes, and assess feasibility in routine hospital settings.

## Funding

This research did not receive any specific grant from funding agencies in the public, commercial, or not-for-profit sectors

## Nagoya protocol statement

Not applicable. This study did not involve the collection or use of genetic resources or fieldwork requiring permits.

## CRediT authorship contribution statement

**Femke van Kessel:** Writing – review & editing, Writing – original draft, Methodology, Formal analysis, Conceptualization. **Harm H.J. van Noort:** Writing – review & editing, Methodology, Investigation. **Minke Reimink:** Writing – review & editing, Investigation, Conceptualization. **Kim Kamphorst:** Writing – review & editing, Supervision, Methodology, Data curation, Conceptualization.

## Declaration of competing interest

The authors declare that they have no known competing financial interests or personal relationships that could have appeared to influence the work reported in this paper.
